# Impact of poly(ethylene glycol) functionalized lipids on ordering and fluidity of colloid supported lipid bilayers†

**DOI:** 10.1039/d2sm00806h

**Published:** 2022-09-27

**Authors:** Emma C. Giakoumatos, Levena Gascoigne, Berta Gumí-Audenis, Álvaro González García, Remco Tuinier, Ilja K. Voets

**Affiliations:** Laboratory of Self-Organizing Soft Matter, Department of Chemical Engineering and Chemistry, Eindhoven University of Technology P.O. Box 513 5600 MB Eindhoven The Netherlands I.Voets@tue.nl; Institute for Complex Molecular Systems (ICMS), Eindhoven University of Technology P.O. Box 513 5600 MB Eindhoven The Netherlands; Laboratory of Physical Chemistry, Department of Chemical Engineering and Chemistry, Eindhoven University of Technology P.O. Box 513 5600 MB Eindhoven The Netherlands

## Abstract

Colloid supported lipid bilayers (CSLBs) are highly appealing building blocks for functional colloids. In this contribution, we critically evaluate the impact on lipid ordering and CSLB fluidity of inserted additives. We focus on poly(ethylene glycol) (PEG) bearing lipids, which are commonly introduced to promote colloidal stability. We investigate whether their effect on the CSLB is related to the incorporated amount and chemical nature of the lipid anchor. To this end, CSLBs were prepared from lipids with a low or high melting temperature (*T*_m_), DOPC, and DPPC, respectively. Samples were supplemented with either 0, 5 or 10 mol% of either a low or high *T*_m_ PEGylated lipid, DOPE-PEG_2000_ or DSPE-PEG_2000_, respectively. Lipid ordering was probed *via* differential scanning calorimetry and fluidity by fluorescence recovery after photobleaching. We find that up to 5 mol% of either PEGylated lipids could be incorporated into both membranes without any pronounced effects. However, the fluorescence recovery of the liquid-like DOPC membrane was markedly decelerated upon incorporating 10 mol% of either PEGylated lipids, whilst insertion of the anchoring lipids (DOPE and DSPE without PEG_2000_) had no detectable impact. Therefore, we conclude that the amount of incorporated PEG stabilizer, not the chemical nature of the lipid anchor, should be tuned carefully to achieve sufficient colloidal stability without compromising the membrane dynamics. These findings offer guidance for the experimental design of studies using CSLBs, such as those focusing on the consequences of intra- and inter-particle inhomogeneities for multivalent binding and the impact of additive mobility on superselectivity.

## Abstract 2

Colloid Supported lipid bilayer membrane fluidity is dependent on the amount of lipopolymers incorporated for stabilization. Beyond a threshold mol fraction of lipopolymers, lateral mobility is significantly reduced due to heterogeneity.

## Graphical abstract

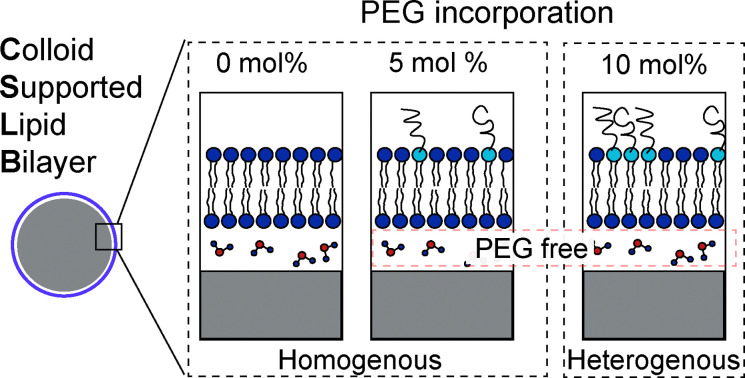

## 1. Introduction

Colloid supported lipid bilayers (CSLBs) are lipid bilayer coatings on the surface of colloidal particles. These form upon the dissolution of small unilamellar vesicles (SUVs), typically comprising zwitterionic phospholipids, into a dispersion of colloidal particles.^1–10^ Interestingly, this strategy yields surface-functionalized colloids without direct chemical modification of the particle surface to introduce reactive groups. Instead, a SUV bilayer carrying membrane-bound additives is transferred onto the particle scaffold to confer attributes such as enhanced stability, fluorescence, and multivalent binding. Examples of such additives include poly(ethylene glycol) (PEG), fluorescent probes, and DNA linkers capable of impacting the interaction and/or specific binding between particles.^5,6,8–11^ All of these additives can be inserted into the membrane *via* conjugation to suitable anchors such as cholesterols and phosphatidylethanolamines.^9,12^ This versatility inspired various groups to exploit the potential of CSLBs as building blocks for designer colloidal materials. For example, colloidal scale joints with tuneable flexibility and shape-shifting aggregates have been created from CSLBs decorated with DNA.^8,9,11^ CSLBs have also been applied as model biological membranes for various applications, such as drug screening, drug delivery, sensing and for studying protein–cell interfacing.^10,13–21^

The formation of CSLBs from the parent SUVs and colloidal particles,^5,13^ such as silica, requires the lipid bilayer to be sufficiently fluid-like to enable the SUVs to rupture, spread and fuse to encapsulate the contacted particle.^1–3,18,22–26^ This is achieved at temperatures above the transition temperature, *T*_m_, of the constituent lipids, which depends markedly on lipid chemical structure, particularly on the tail length and carbon saturation.^27^ In mixed membranes of immiscible lipids, the fluidity may further be exploited to generate spatiotemporal variations in composition due to phase separation.^28–30^ Patchy particles with considerable variation in patch size and number have been prepared in this manner.^31^ This potential to tune additive incorporation, mobility, and reconfiguration makes CSLBs an appealing building block for creating colloidal materials. Homo- and heterogeneous distributions of (multiple) surface mobile and immobile linkers are accessible.^32^ These features offer exciting opportunities to develop systems with built-in reconfigurability^33^ to investigate the consequences of intra- and inter-particle inhomogeneities on multivalent binding and the impact of additive mobility on phenomena such as superselectivity.^34^

To achieve directed assembly of CSLB building blocks through specific, short-range attractive interactions between (mobile) complementary membrane-bound (DNA) linkers,^9,11,35^ strong and aspecific attractions must be suppressed. These would otherwise interfere with and compromise the predictability and programmability of the colloidal assembly process. Lipids bearing neutral polymer chains, typically poly(ethylene glycol) (PEG), are commonly introduced within CSLBs for this purpose. PEG chains of 2 kDa or larger on PEGylated lipids undergo steric exclusion from large unilamellar vesicles (LUV) and are of sufficient length to sterically stabilize LUVs.^35,36^ Lattice computations demonstrated that PEG chains protruding from the outer leaflet effectively reduce the weak attractive forces^11,35^ which act between phospholipid bilayers.^37^ Similarly, a sufficiently high amount of PEG-conjugated lipids with sizeable PEG chains will promote steric repulsion between CSLBs in close proximity.

Incorporating stabilizers such as PEGylated lipids to achieve colloidal stability must be done with care. The appealing signature features of CSLBs, such as their ability to switch between a fluid-like and solid-like state, should not be compromised. Previous work by others on planar and colloid-supported bilayers established that the impact of polymers on bilayers is complex and multifactorial.^9,38–40^ Between supported bilayers and their solid substrates, there is a thin 10–30 Å water layer, which acts as a lubricant to reduce the frictional coupling between the support and the bilayer and weakens the impact of the substrate on the bilayer.^41,42^ Recently, Beckers *et al.*^43^ compared membrane fluidity between unsupported giant unilamellar vesicles (GUVs), planar and colloidal supported bilayers. The influence over substrate-bilayer interacts has no influence over lipid packing, however does have a significant impact on membrane fluidity, with a significant decrease in diffusion when added to planar support, and even further when added to CSLB. Therefore, a significant amount of work has focused on incorporating PEG within the planar supported lipid membrane *via* PEGylated lipid^44–46^ or PEG tethered to substrates^39,40^ in order to decrease substrate-bilayer interactions, thus increasing membrane fluidity equal to a unsupported membrane. Increases to lipid fluidity have been specifically shown on planar substrates at concentrations <4 mol% of PEGylated lipids.^47^ Rinaldin *et al.*^9^ demonstrated that incorporation of low PEGylated lipids (1–2 mol% <3 kDa) into CSLB do not significantly increase lipid diffusion of the bilayer. Further increases to PEG incorporation resulted in complete loss of fluidity and membrane inhomogeneity which was attributed to hampered spreading and fusion during CSLB formation and stabilizer pinning onto the spherical supports *via* PEG adsorption.

In this contribution, we critically evaluate the impact on lipid ordering and membrane fluidity of the insertion of stabilizers into 3 μm-sized silica CSLBs in the form of PEGylated lipids. We examine whether the inserted amount and lipid anchor type affect lipid ordering by differential scanning calorimetry (DSC) and membrane fluidity by fluorescence recovery after photobleaching (FRAP). To this end, we purposely incorporated sizeable PEG_2000_ chains within solid-like (DPPC; *T*_m_ 41 °C) and liquid-like (DOPC, *T*_m_ −17 °C) CSLBs through solid-like (DSPE-PEG_2000_, *T*_m_ 71 °C based on DSPE anchor) and liquid-like (DOPE-PEG_2000_, *T*_m_ −16 °C based on DOPE anchor) lipid anchors, respectively.^48^ We find that incorporation of up to 5 mol% of DOPE-PEG_2000_ and DSPE-PEG_2000_, both commonly employed to enhance colloidal stability, did not profoundly impact lipid ordering nor the fluidity of lipid bilayers. Neither the silica scaffold nor the PEGylated lipid affected the lipid phase transition temperature or the timescale of fluorescence recovery significantly. By contrast, the fluidity of the liquid-like DOPC membranes was markedly reduced upon incorporating 10 mol% of both PEGylated lipids. Self-consistent mean-field theory (SCFT) computations were performed aiming to relate these observations to the distribution of the pristine and PEGylated lipids within the CSLBs and parent SUVs. The results suggest that the marked reduction in mobility is not due to the binding of PEG to the silica support; instead, the PEGylated lipids reside primarily in the outer leaflet, with the PEG chains protruding into the solution. The herein reported experimental and computational findings offer guidance for the experimental design of systematic studies on CSLBs aiming to investigate or exploit the effect of membrane fluidity on particle interactions, structuration and other phenomena.

## 2. Experimental section

### 2.1. Materials and preparation

#### 2.1.1. Materials

1,2-Dioleoyl-*sn-glycero*-3-phosphocholine (DOPC), 1,2-dipalmitoyl-*sn-glycero*-3-phosphocholine (DPPC), 1,2-dioleoyl-*sn-glycero*-3-phosphoethanolamine (DOPE) and 1,2-distearoyl-*sn-glycero*-3-phosphoethanolamine (DSPE), 1,2-distearoyl-*sn-glycero*-3-phosphoethanolamine-*N*-[methoxy(polyethylene glycol)-2000] (ammonium salt) (DSPE-PEG_2000_), 1,2-dioleoyl-*sn-glycero*-3-phosphoethanolamine-*N*-[methoxy(polyethylene glycol)-2000] (ammonium salt) (DOPE-PEG_2000_) were purchased from Avanti lipids *via* Sigma Aldrich. Additionally 1,2-dioleoyl-*sn-glycero*-3-phosphoethanolamine-*N*-(lissamine rhodamine B sulfonyl) (DOPE–rhod) and 1,2-dipalmitoyl-*sn-glycero*-3-phosphoethanolamine-*N*-(lissamine rhodamine B sulfonyl) (DPPE-rhod) used for confocal imaging where obtained from Avanti lipids *via* Sigma Aldrich. All solvents and salts, (HEPES buffer, NaCl, chloroform) used for liposome hydration were purchased from Sigma Aldrich. 3.0 ± 0.14 μm silica particles (SiO_2_-R-L3628) were obtained from Microparticles GmbH.

### 2.2. Methods

#### 2.2.1. Preparation of small unilamellar vesicles

Briefly, 1 mM small unilamellar vesicles (SUVs) of 1,2-dioleoyl-*sn-glycero*-3-phosphocholine (DOPC) or 1,2-dipalmitoyl-*sn-glycero*-3-phosphocholine (DPPC) were formed *via* the thin-film method and hydrated using 10 mM HEPES and 50 mM NaCl buffer at pH 7.4 at 20 °C (DOPC) and 60 °C (DPPC) to ensure hydration above the melting temperature of the primary lipid. Hydrated samples were then extruded using an Avanti Mini Extruder (Avanti Polar Lipids, USA) 20 times with a 200 nm filter and 21 times with a 100 nm filter while maintained above *T*_m_ of the phospholipids. Further details are described in the ESI.†

#### 2.2.2. Preparation of colloid supported lipid bilayers (CSLBs)

CSLBs were formed *via* the mixing and deposition of 1 mM SUVs onto 3 μm silica particles in 10 mM HEPES with 50 mM NaCl at 20 °C (DOPC) and 60 °C (DPPC) to ensure deposition above the melting temperature of the primary lipid.^25^ After 1 hour, samples were washed *via* centrifugation 5 times, followed by 1 hour sonification. CSLB was stored at 4 °C and analyzed within one week.

#### 2.2.3. Preparation of fluorescent CSLBs

To provide fluorescence to the bilayer, dye-conjugated phospholipid, with phase behaviour similar to the primary lipid, was included in the lipid mixture when preparing the thin film for SUV formation. Dye-conjugated phospholipids with a *T*_m_ similar to the majority lipid were selected. Specifically, 0.25 mol% of 1,2-dioleoyl-*sn-glycero*-3-phosphoethanolamine-*N*-(lissamine rhodamine B sulfonyl) (DOPE-rhod, *T*_m_ = −16 °C based on DOPE anchor) for DOPC and 0.25 mol% 1,2-dipalmitoyl-*sn-glycero*-3-phosphoethanolamine-*N*-(lissamine rhodamine B sulfonyl) (DPPE-rhod, *T*_m_ = 74 °C based on DPPE anchor) for DPPC were used.

#### 2.2.4. Differential scanning calorimetry (DSC)

Calorimetric studies were carried out using a Multi-Cell micro-DSC (TA Instruments). DPPC colloid supported lipids and SUV samples, 8 mM in concentration, were dispensed into pre-calibrated sample cells (∼400 mg). DOPC samples were outside the achievable temperature range (−10 to 130 °C, TA), and no visible *T*_m_ was apparent as expected. Additionally, both buffer solution and silica particles suspended in buffer were measured simultaneously for background subtraction for SUV and CSLB samples, respectively. A scan heating rate and cooling rate of 0.5 °C min^−1^ and 1 °C min^−1^ were used for all samples, respectively. Sample runs were performed in the temperature range between 10 °C and 80 °C and repeated in technical triplicate to ensure repeatability. Acquisition and analysis were completed on NanoAnalyze Data Analysis (Version 3.11.0, https://www.tainstruments.com) and Origin software. The baseline of raw data was constructed *via* sigmoidal baseline fitting, followed by Gaussian fitting models to ascertain *T*_m_. Data were analyzed above 20 °C to exclude the initial heating ramp. Additionally, samples exhibited no thermal events over the temperature range between 50 °C and 80 °C and therefore, temperature ranges between 20 °C and 60 °C are shown herein. All data shown in thermographs in manuscript are taken from the second heating scan. Background subtracted full heating and cooling scans shown in Fig. S10 (ESI†).

#### 2.2.5. Fluorescence recovery after photobleaching (FRAP)

Confocal FRAP analysis was performed on Leica SP8 inverted microscope with a 100× oil immersion objective (HC PL APO CS2 100×/1.40 OIL). Images (512 × 512 pixels) were recorded in the unidirectional scan mode at 600 Hz and a pinhole at 1 Airy unit (151 μm). Samples were excited with an argon laser at 552 nm, and emission signals were collected in 565–656 nm bands. Transmission and detector gain were optimized for each sample to minimize bleaching effects.

Microscopy slides were pre-treated with a poly-lysine solution for 15 minutes to prevent lipid adsorption and increase the stabilization of particles. After 15 minutes, slides were washed in triplicate with Milli-Q-water followed by CSLB samples and immediately imaged. Briefly, singlets (*i.e.*, not aggregated particles) were identified and imaged at 0.5–5% laser intensity, and a small region of interest of the particle was selected for bleaching for 1–2 seconds at 100% laser intensity (ESI† S4). Post bleaching samples were imaged for 10 minutes at the same laser intensity pre-bleach state. Images were analyzed using ImageJ to generate recovery curves. Any apparent drift in particles was corrected using StackReg plugin within ImageJ.^49^

All fluorescence data were normalized (*f*_(*t*)_) as described by Michul Kang^50^ using eqn (1);1
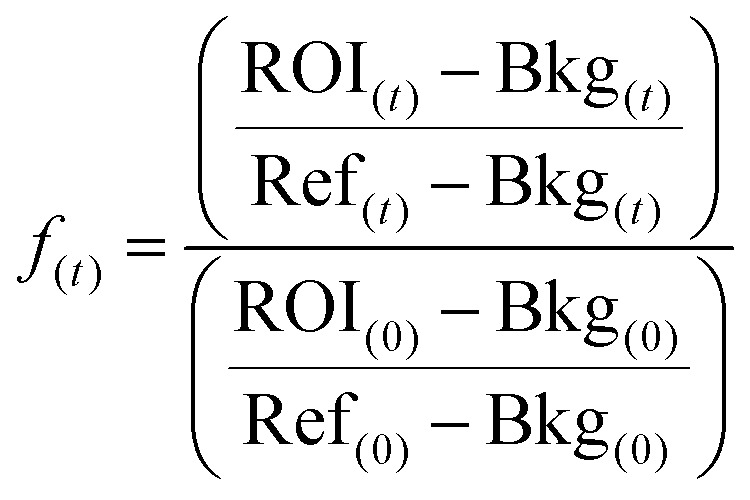
where the Ref and Bkg are the fluorescence of the reference area and background (60 × 60 pixel) of an image, respectively. The FRAP region of interest, ROI, was taken at 15 × 15 pixel size. After normalization, the average of all samples was taken with a minimum sample size of 9.

Samples were fitted using the following expression;2
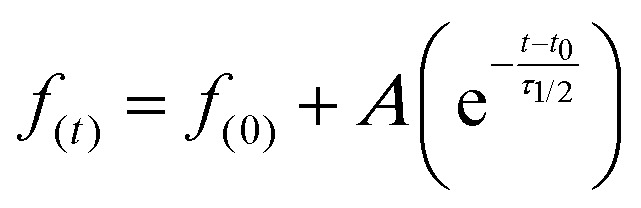
where *f*_(0)_ is the initial normalized intensity, *A* is te extent of recovery, *t − t*_0_ is the time elapsed since initial recovery and *τ*_1/2_ the half life recovery time.

#### 2.2.6. Self-consistent mean-field theory (SCFT)

**Fig. 1:**
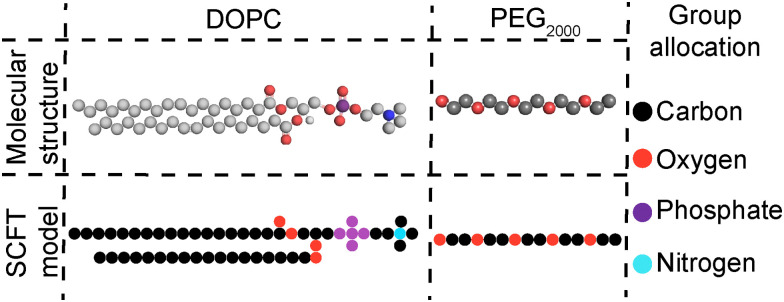
SCFT coarse-graining of the molecular structures of DOPC and short PEG_2000_ chains; groups presented in the rightmost column.

Self-consistent mean-field theory (SCFT)^51–53^ computations were performed on DOPC membranes to examine the location of PEG within (supported) bilayers. SCFT is a theoretical approach involving a lattice discretization scheme, which can be used to predict the equilibrium morphology and spatial composition of self-assembled structures by considering each unimeric building block as a set of chemical units. Based on previous works, each chemical unit of the lipid was considered as taking up a single lattice unit, except for phosphate, which takes five units arranged in a cross-like shape (Fig. 1).^54,55^ SCFT was performed on DOPC SUVs and CSLBs but not on DPPC membranes, since SCFT is principally predictive for liquid components. Further details on the SCFT computations can be found in the ESI.†

## 3. Results and discussion

### 3.1. Impact of silica substrate on lipid ordering and melting cooperativity in DPPC membranes

**Fig. 2:**
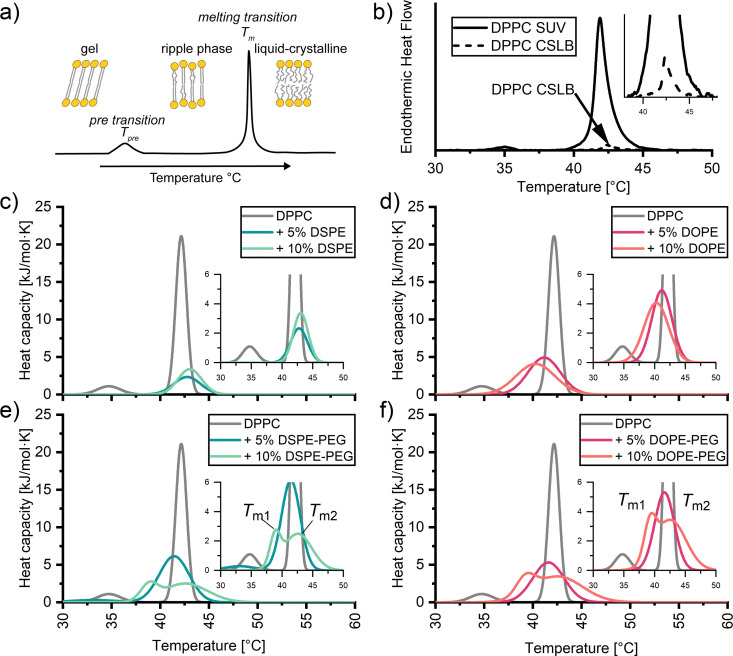
(a) Scheme of differential scanning calorimetry (DSC) profiles with associated distinguishable peaks of the transitions within the membrane from the gel to ripple phase (*T*_pre_), and to the liquid-crystalline phase (*T*_m_). DSC thermograms of (b) DPPC SUVs and DPPC on a 3 μm silica particle scaffold. Endothermic heat flow is present due to the inability to convert CSLB data to heat capacity accurately. DSC thermograms of DPPC SUVs with 5 and 10 mol%; (c) DSPE and (d) DOPE, (e) DSPE-PEG_2000_ and (f) DOPE-PEG_2000_ incorporated into the membrane.

A convenient method to identify the impact of the silica scaffold and added PEGylated lipids on the lipid membrane is differential scanning calorimetry (DSC). With DSC we can detect the phase transition of lipids, such as the pre-transition to the ripple phase (*T*_pre_) and the transition from the gel to liquid-crystalline state (*T*_m_) (Fig. 2a).^56,57^ The transitions are signalled by a characteristic peak from which we can determine an excess enthalpy, transition temperature and full width half maximum (FWHM). The latter can be taken as a qualitative measure of the cooperativity of melting of the hydrocarbon chains during the melting transition. A strong, sharp FWHM close to 1 indicates that the entire lipid membrane melts in a more or less concurrent manner. In contrast, increase in FWHM suggest the presence of defects within the membrane, causing different regions within the membrane to melt at slightly different temperatures. As expected, the thermograms of SUVs composed of DPPC without PEGylated lipids exhibit two distinct peaks, *T*_pre_ 34.85 ± 0.12 °C and *T*_m_ 42.12 ± 0.11 °C (Fig. 2b, solid). Unexpectedly, the heat effects were less defined for the corresponding CSLB samples (Fig. 2b, dash). We no longer observe a *T*_pre_ and can crudely observe a *T*_m_ of 42 °C for the CSLBs. The decrease in peak height is tentatively attributed to a reduced lipid concentration in these samples. A sharp and pronounced *T*_m_ peak was observed previously by others for smaller (640 nm) CSLBs,^58^ indicating that the silica scaffold itself has a negligible impact on the temperature and cooperativity of the melting transition. Therefore, the impact of anchor lipids and PEG on the membranes was studied hereafter by DSC experiments on the unsupported SUVs bilayers.

### 3.2. Impact of anchor lipids on lipid ordering and cooperativity in DPPC membranes

Next, DSC was employed to examine if DOPE and/or DSPE anchors impact lipid ordering in DPPC membranes in SUVs (Fig. 2c and d). The results presented in Fig. 2c and d reveal no significant differences in *T*_m_ regardless of the amount and nature of the incorporated anchor. Instead, the melting temperatures ranged from 40 °C to 42 °C for all SUVs carrying lipid anchors. Apparently, the gel-like anchor DSPE (*T*_m_ 74 °C) shifts the *T*_m_ of the DPPC membrane +1 °C regardless of incorporation percentage. Whereas the fluid-like anchor DOPE (*T*_m_ −16 °C) induces a slight downward shift in *T*_m_, with −1 °C per 5% anchor incorporation. Differences of *T*_m_ peak height between DSPE and DOPE samples have been attributed to variation in concentration during preparation.

Whilst the anchor has little effect (±2 °C) on the position of the most pronounced peak in the thermograms, its width is significantly broadened in all cases (Fig. 2c and d). Such an increase in the FWHM has been related to defects within the pure membrane.^57^ Here these are presumably due to the compositional differences between the head groups and tails of the anchors and DPPC. It has been reported^59–61^ that such compositional can cause coexistence of gel like and liquid like phases. The coexistence of two different phases renders the melting transition less cooperative.

### 3.3. Impact of PEGylated lipids on lipid ordering and melting cooperativity in DPPC membranes

To better understand why an increase in the stabilizer fraction within CSLBs can decrease membrane fluidity as determined by Rinaldin *et al.*,^9^ we examined the influence of PEG stabilizer incorporation on the phase behaviour of DPPC membranes *via* DSC (Fig. 2c and d). Similarly to lipid anchors, we find that the *T*_m_ is mildly affected by the nature of the incorporated stabilizer. For both 5 mol% DOPE-PEG and DSPE-PEG stabilized samples decreased *T*_m_ to 41 °C. Intriguingly, incorporating 10 mol% of stabilizers causes peak splitting. This peak splitting is due to two endothermic transitions within the membrane, thus producing two different melting temperatures. This could signify the saturation point of PEGylated lipids within the membrane, causing a transition from a lamellar to a micellar state.^62,63^ However, we find no indication of free PEG (hydrodynamic radius >15 nm) or micelle like structures (hydrodynamic radius 15–30 nm^64^) *via* dynamic light scattering (ESI†). Therefore we tentatively attribute peak splitting to clustering of the PEGylated lipids causing lateral phase separation.^64,65^ This is likely induced by the PEG chains, since peak splitting is absent in the thermograms of the SUVs with anchors only (*i.e.*, without PEG). PEG clustering within lipid membranes has previously been observed in planar supported bilayers *via* atomic force microscopy and fluorescence imaging,^60^ and in liposomes *via* atomic force osmotic stress studies.^66,67^ In sum, DSC demonstrates that the solid-like to fluid-like transition in DPPC SUVs and CSLBs occurs in the temperature range between 40 °C and 43 °C irrespective of the amount of stabilizer and anchor type for up to 10 mol% of DOPE-PEG_2000_ and DSPE-PEG_2000_. Furthermore, elevated amounts of stabilizer induce defects and at 10 mol% inhomogeneities within the membrane.

### 3.4. Impact of PEGylated lipids on the fluidity of colloid supported bilayers of DOPC and DPPC

**Fig. 3:**
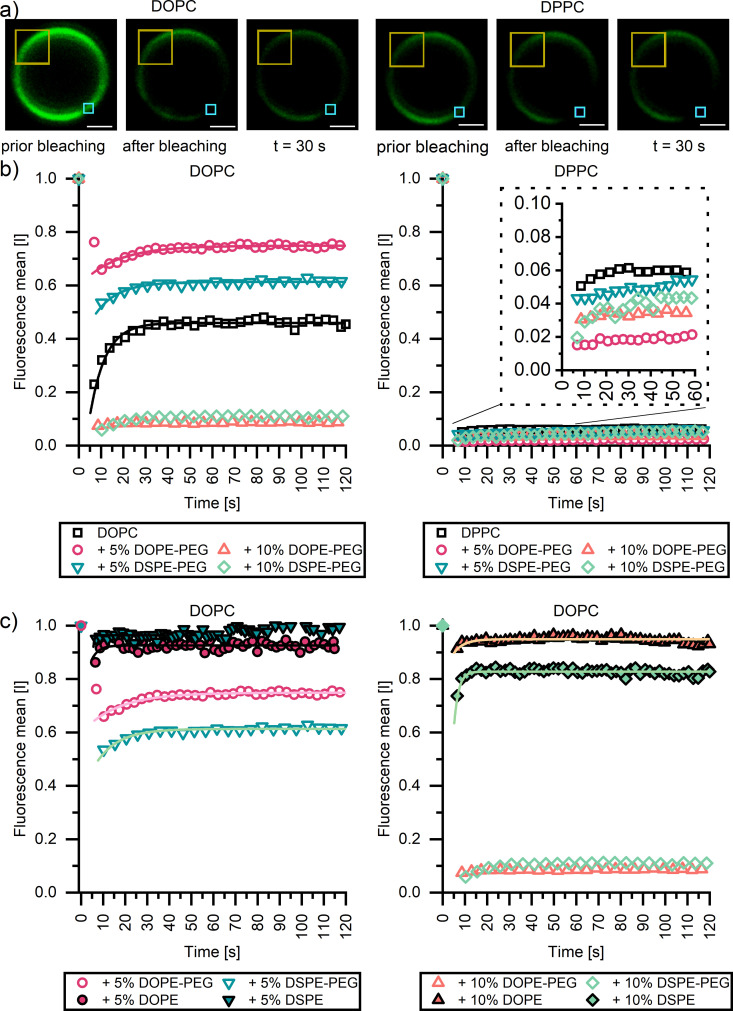
Fluorescence recovery after photobleaching of (a) (left panel) DOPC CSLBs labelled with 0.25 mol% DOPE-rhodamine and (right panel) DPPC CSLBs labelled with 0.25 mol% DPPE-rhodamine prior to bleaching, immediately following bleaching and at 30 s respectively. The blue and yellow boxed areas highlight the bleached ROI and reference ROI used to analyze the FRAP recovery curve, respectively. Scale bar 1 μm (b) recovery of fluorescence over the time for CSLBs of DOPC (left) and DPPC (right), with 0, 5 and 10 mol% DOPE-PEG_2000_ or DSPE-PEG_2000_. (c) Recovery of fluorescence over the time for CSLBs of DOPC with 5 mol% (left) and 10 mol% (right) of DOPE, DSPE, DOPE-PEG_2000_ or DSPE-PEG_2000_. Symbols show the mean FRAP data averaged over a minimum of 9 experiments.

To investigate whether the inclusion of PEGylated lipids through low or high *T*_m_ lipid anchors impacts membrane fluidity, fluorescence recovery after photobleaching (FRAP) experiments were undertaken using confocal microscopy (Fig. 3). Herein, a small, defined region is bleached (red boxed area, Fig. 3a), and the recovery of the fluorescence intensity in the corresponding area is monitored over time (Fig. 3b). We anticipated a markedly different response for fluid-like DOPC membranes, wherein bleached lipids and unbleached dye-conjugated lipids can redistribute due to translation, and solid-like DPPC membranes, in which lateral mobility is prohibited. Indeed, as expected, DOPC membranes (partially) recover their fluorescence, as indicated by the presence of a plateau in fluorescence (*F*_∞_). The speed of recovery to half of *F*_∞_, half life (*τ*_1/2_), was 7.00 ± 0.24 s. By contrast, no fluorescence recovery was observed for the DPPC–based CSLBs within the experimental window of 10 minutes. We, therefore, report for Fig. 3 data up to 120 seconds (see Fig. S2 (ESI†) for full-time scale image).

Inserting 5 mol% or 10 mol% of PEGylated lipids did not markedly increase DPPC membrane fluidity. No fluorescence recovery was observed for any of the DPPC bilayers with or without the stabilizers indicating that the ordered gel nature of the DPPC membrane remained. By contrast, the incorporation of both PEGylated lipids had a profound impact on the fluidity of the DOPC CSLBs (Fig. 3b left panel). Membranes with up to 5 mol% of PEGylated anchor remained highly mobile, with *τ*_1/2_ of 16.20 ± 1.5 and 10.10 ± 0.74 for DOPE-PEG and DSPE-PEG, respectively. However, bilayers with 10 mol% of either PEGylated anchor did not recover at all. Instead, the signal of the bleached area remained as low as directly after bleaching, just as we observed for the DPPC CSLBs with a far higher *T*_m_. This trend is in agreement with previous planar supported membranes^44,46^ and CSLB studies.^9^

Interestingly, it appears that only small amounts (<5 mol%) of PEGylated lipids can be inserted into CSLBs without compromising lateral mobility, even if lipid anchors with a low *T*_m_ are employed. To examine whether PEG_2000_ stabilizers are the culprit (*i.e.*, whether the reduction in fluidity is related to the PEG chains rather than the lipid anchor), FRAP experiments were also conducted on CSLBs with 5 mol% and 10 mol% of either DSPE or DOPE (*i.e.*, without the pendant PEG_2000_). Notably, these CSLBs appear more mobile as the pristine DOPC CSLBs exhibiting a rapid recovery, with a *F*_∞_ < 10 seconds (Fig. 3c). Due to the apparent rapid recovery, initial key data points after recovery were not recorded, therefore difficult to accurately determine *τ*_1/2_ of samples. Similarly, to our findings, Fernado *et al.*,^45^ found no differences in FRAP recovery in DOPC planar supported samples with variations of PEGylated anchors.^45^ This shows that tail length and carbon saturation of lipid anchor have no noticeable effect on the fluidity of supported membranes. Therefore, the reduction in FRAP recovery of the CSLBs with 10 mol% PEGylated lipids has to do with the incorporated PEG chains and may be related to the phase separation of the lipopolymers (which was not directly observable *via* CLSM imaging), which increases the heterogeneity of the membrane and compromises its collective response. Both of these suggestions are not directly observable *via* CLSM imaging. Gaining further mechanistic insight will require selective, single-molecule tracking experiments of the mobility of the majority lipids and the PEGylated lipids within the mixed membranes, so as to monitor translation of the individual components at high spatiotemporal resolution instead of probing ensemble responses averaged over multiple species and locations within a more or less heterogeneous membrane.

In line with previous reports on LUVs,^35,62^ planar SLBs^45,46,66^ and CSLBs,^9^ our DSC and FRAP experiments demonstrate that the inclusion of 10 mol% of PEG_2000_ chains significantly impacts lipid ordering and reduces membrane fluidity. Some of these studies tentatively attributed the loss of mobility to adsorption of PEG onto the silica surface.^9,38^ This is at odds, however with neutron and X-ray reflectivity studies on planar SLBs, which revealed that the PEG chains of PEG conjugated lipopolymers do not yield a hydrated cushion beneath the bilayer unless they are covalently tethered by reactive terminal ends to bind permanently to the underlying solid support.^39^ To investigate if PEG is likely to adsorb at the silica particles and pin the bilayer or rather reside on the exterior of the CSLBs, we turned to self-consistent field computations.

### 3.5. Theoretical prediction of lipopolymer location in colloid supported DOPC bilayers

**Fig. 4:**
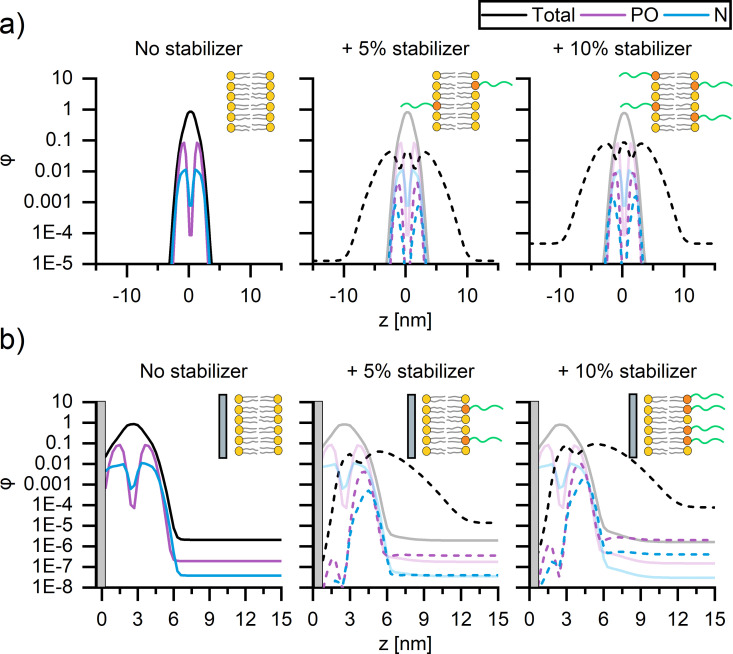
Self-consistent mean-field theory predictions of the concentration profiles of DOPC (full lines) SUVs and CSLBs with 0, 5 mol% and 10 mol% DSPE-PEG_2000_ (dotted lines). The average spatial composition profiles and corresponding schematic representations of (a) SUVs and (b) CSLBs composed of DOPC with 0 (left), 5 mol% (middle) and 10 mol% (right) DSPE-PEG_2000_ stabilizer. The silica surface in b is represented by the grey region.

To better understand the origin of the impact of PEG on membrane fluidity, self-consistent mean-field theory (SCFT)^51–53^ computations were performed to examine the location of PEG within (supported) bilayers. Since SCFT is principally predictive for liquid components, we focus on the trends exhibited by modelling the interactions of the liquid-like DOPC and not gel-like DPPC membranes of SUVs and CSLBs, with and without DSPE-PEG_2000_ (Fig. 4).

We compute the concentration profiles of phosphate, nitrogen, and the total lipid molecule that quantify the bilayer cross–section for both SUVs (Fig. 4a) and CSLBs (Fig. 4b) with 0, 5 mol% and 10 mol% DSPE-PEG_2000_, allowing for a direct comparison of the location of the hydrophobic tails, the hydrophilic heads, the polymer chain of the lipopolymers within the lipid bilayer and the center of the bilayer. The resulting density profiles display the local component volume fraction *φ* as a function of the position *z*, with *z =* 0 corresponding to the center of the bilayer for SUVs (Fig. 4a) and the silica surface for CSLBs (Fig. 4b). The spatial composition of all SUVs, with and without DSPE-PEG_2000_, were symmetric, with similar average contents of DOPC and DSPE-PEG_2000_ in the two leaflets, regardless of the DSPE-PEG_2000_ concentration (Fig. 4a). The DOPC bilayer was found to be ∼5 nm in thickness, in accord with the experimental thickness determined by atomic force microscopy (Fig. S6, ESI†). The PEGylated lipid extends from the inner and outer leaflets of the DOPC bilayer into solution providing steric stability.

Next, the spatial composition of CSLBs composed of DOPC with and without DSPE-PEG_2000_ was examined by considering the equilibrium morphology of bilayers in proximity to a silica surface (Fig. 4b). In proximity to the silica surface located now at *z =* 0, the bilayer without PEGylated lipids remained primarily symmetric as expected (Fig. 4b, left). However, on closer inspection, the nitrogen head group (blue) of the DOPC appears to be contiguous to the silica surface. The DSPE-PEG_2000_ profiles (Fig. 4b, middle, right) exhibit a striking asymmetry with the majority (small amount of PEGylated lipids remain within inner leaflet) of the PEGylated lipids residing in the outer leaflet of the bilayer and extending into solution. We speculate that this is because PEG chains of the lipopolymers within the water layer between the bilayer and silica surface are conformationally confined, which is entropically unfavourable. In line with previous SCFT adsorption studies,^68,69^ we theorize the lipids have a stronger affinity to silica than that of PEG. It is likely the PEG free portion of lipid membrane displace majority of adsorbed PEGylated lipids. The strong asymmetry observed in the SCF computations suggests that the lipopolymer promotes colloidal stability and, furthermore, that it is unlikely that binding of PEG onto the silica surface causes the reduction of bilayer mobility upon insertion of 10 mol% DSPE-PEG_2000_.

## 4. Conclusion

Colloid supported lipid bilayers were prepared by deposition onto 3 μm-sized silica particles of small unilamellar vesicles composed mainly of DOPC or DPPC and supplemented with PEG_2000_ for stabilization. Differential scanning calorimetry and fluorescence recovery after photobleaching experiments were performed to investigate whether the solid support, the abundance and the anchor type of PEGylated lipids within the CSLBs impacted lipid ordering and membrane fluidity. Surprisingly, DSC on DPPC membranes showed that the solid support and lipid linker composition had little impact on the *T*_m_ of samples. However, at 10 mol% PEG_2000_ incorporation, the overall membrane cooperativity is reduced due to the clustering of PEG chains. FRAP revealed that the solid-like nature of the DPPC membrane was retained under all conditions. In contrast, the fluid-like nature of the DOPC membranes was lost upon incorporating 10 mol% of either DOPE-PEG_2000_ or DSPE-PEG_2000_. Notably, mobility was recovered for membranes supplemented with either DOPE or DSPE, even at 10 mol% as long as these were not conjugated to PEG_2000_. Numerical SCFT lattice computations were employed to predict the concentration profiles of the DOPC and DSPE-PEG_2000_ lipids within the bilayers. While the membrane composition of SUVs was found to be symmetric, displaying similar average amounts of DOPC and DSPE-PEG_2000_ lipids in both leaflets, regardless of the amount of inserted lipopolymer, the presence of silica initiated a noticeable asymmetry. In the CSLBs, DSPE-PEG_2000_ resides primarily in the outer leaflet. This asymmetric configuration is favourable in terms of colloidal stability. It suggests that PEG binding to silica is not the origin for the pronounced reduction in bilayer mobility in DOPC CSLBs with 10 mol% DSPE-PEG_2000_. Rather, the reduction in bilayer mobility is caused by the outer leaflet experiencing the effect of almost 20 mol% PEGylated lipids, and likely to be causing phase separation of lipids through PEG clustering.

The herein reported experimental and computational findings offer guidance for the experimental design of systematic studies on CSLBs aiming to investigate or exploit the effect of membrane fluidity under isothermal conditions on the specificity and efficiency of particle binding, phase behaviour, and directed assembly. Membrane fluidity, and concomitantly the lateral mobility of stabilizers and linkers, is dependent on the amount of lipopolymers incorporated for stabilization. Beyond a threshold mol fraction of lipopolymers, lateral mobility is significantly reduced due to heterogeneity. Disadvantageously, this loss of mobility cannot be circumvented rigorously using lipid anchors with a transition temperature comparable to that of the majority lipid within the CSLB. Instead, it is the degree of lipopolymer incorporation that should be customized.

## Author contributions

EG & IKV conceived the experiments. EG, LG performed the experiments and the data analysis. BGA performed exploratory experiments. AGG performed and analyzed the SCFT computations. IKV and RT supervised the research. EG, LG and IKV wrote the manuscript with contributions from all authors.

## Conflicts of interest

There are no conflicts to declare.

## Supplementary Material

SM-018-D2SM00806H-s001
