# Supplementary material for: Impact of poly(ethylene glycol) functionalized lipids on ordering and fluidity of colloid supported lipid bilayers
Source: Soft Matter. 2022 Sep 27;18(39):7569–78. doi: 10.1039/d2sm00806h (PMC9555145; doi:10.1039/d2sm00806h)
Supplement: SM-018-D2SM00806H-s001 [file SM-018-D2SM00806H-s001.pdf]

Electronic Supplementary Material (ESI) for Soft Matter.
This journal is © The Royal Society of Chemistry 2022

Impact of poly(ethylene glycol) functionalized lipids on ordering and fluidity of colloid supported lipid bilayers

Electronic supplementary information

Emma C. Giakoumatos, [¥1,2,3] Levena Gascoigne,[¥1,2] Berta Gumi Audenis,[1,2] Álvaro González García,[2,3,] Remco Tuinier,[2,3] Ilja K. Voets[*1,2]

[¥] Equal contributions

[*]Corresponding author

[1] Laboratory of Self-Organizing Soft Matter, Department of Chemical Engineering and Chemistry, Eindhoven University of Technology, P.O. Box 513, 5600 MB Eindhoven, The Netherlands
[2] Institute for Complex Molecular Systems (ICMS), Eindhoven University of Technology, P.O. Box 513, 5600 MB Eindhoven, The Netherlands
[3] Laboratory of Physical Chemistry, Department of Chemical Engineering and Chemistry, Eindhoven University of Technology, P.O. Box 513, 5600 MB Eindhoven, The Netherlands

Table of Content

S1. Preparation of silica particles

Figure S1. SEM on purchased 3 µm silica particles

S2. Synthesis of small unilamellar vesicles

S3. Formation of colloid supported lipid bilayers

S4. Fluorescence recovery after photobleaching

Figure S2. FRAP measurement was conducted by isolating a single CLSB particle in the left-hand corner of frame (left). FRAP occurred by isolating a small fraction of bilayer (red box) at 30 x 30-pixel ROI and zoomed in at 48 zoom. Once zoomed, laser intensity was increased to 100% for 2-3 frames (Middle). Laser intensity was than decreased to pre-FRAPed intensity and returned to original zoom factor and monitored for 10 minutes (right). FRAP analysis conducted on CSLB was conducted with the reference and background fluorescence of image with an 60 x 60-pixel area shown in yellow and purple respectively. The ROI monitored for FRAP recovery was 15 x 15-pixel area of the FRAP area of image shown in blue. Scale bar 1 µm.

Figure S3. Full 10-minute scan of FRAP recovery of samples

Figure S4. FRAP recovery curves of DOPC samples with standard deviation included (minimum n=9)

Figure S5. FRAP recovery curves of DPPC samples with standard deviation included (minimum n=9)

S5. Self-consistent mean-field theory (SCFT) predications of the composition of colloid supported lipid bilayers

Figure S6. Grand potential Ω versus number of DOPC surfactants n_{DOPC} with increasing membrane additive DSPE-PEG$_{2000}$ ($n_{DSPE-PEG_{2000}}$) as calculated by SCFT.

Figure S7. SCFT coarse-graining of the molecular structures of DOPC and short PEG$_{2000}$ chains; groups presented in the rightmost column.

Table S1. Different chemical units modelled as lattice beads in SCFT as well as their Flory–Huggins interaction parameter χ_{ij}, relative permeabilities ϵ and charge per bead ν. All beads occupy a single lattice site, except for PO which takes 5 lattice units in a cross-like shape. The two values of N arise from the fact that in DOPC N is positively charged, while in DSPE–PEG$_{2000}$ it is neutralized.

Table S2. SCFT computed equilibrium properties of self-assembled SUVs modelled as bilayers from the unimers specified: aggregation number n^{exc}, bulk unimer concentration (SCFT-provided cmc, ϕ^{bulk}), and gyration (D_g) and hydrodynamic (D_h) diameters.

Figure S8. Equilibrium properties of SUVs emerging from mixing DOPC and DSPE-PEG$_{2000}$ lipids at the indicated ratios where $\alpha = n^{exc}_{DSPE-PEG_{2000}}/(n^{exc}_{DOPC} + n^{exc}_{DSPE-PEG_{2000}})$ a) aggregation number change in DOPC, b) variation on the lipid bulk concentration, c) energy gain per lipid upon self-assembly.

S6. Atomic force microscopy on planar supported lipid bilayers

Figure S9. AFM topography and profile of planar supported lipid bilayers at room temperature of a) DOPC and b) DPPC.

S7. Differential Scanning Calorimetry

Figure S10. DSC thermograms heating (red) and cooling scans (blue) of DPPC SUVs with 0, 5 and 10 mol%; DOPE–PEG$_{2000}$, DSPE–PEG$_{2000}$, DSPE and DOPE incorporated into the membrane. DSC thermograms are of background subtracted raw data.

Figure S11. a) Average T_m of CSLB samples and b) Average full width half maximum of T_m peaks of DPPC SUVs with 5 and 10 mol%; DSPE, DSPE–PEG$_{2000}$,DOPE and DOPE–PEG$_{2000}$ incorporated into the membrane. (n=3)

S8. Dynamic light scattering

Figure S12. Dynamic light scattering curves at 90° of DPPC and DOPC SUV samples with 10%DSPE-PEG incorporated into membrane.

S9. References

S1. Preparation of silica particles

Silica particles of 3 µm were purchased from Micromod GmbH and washed 10 times with milli-Q water by centrifuging and redispersion to remove the storage buffer. Scanning electron microscopy (SEM) imaging found the particles to be spherical and monodisperse with an average diameter of 3.24 ± 0.12 µm (Figure S1).

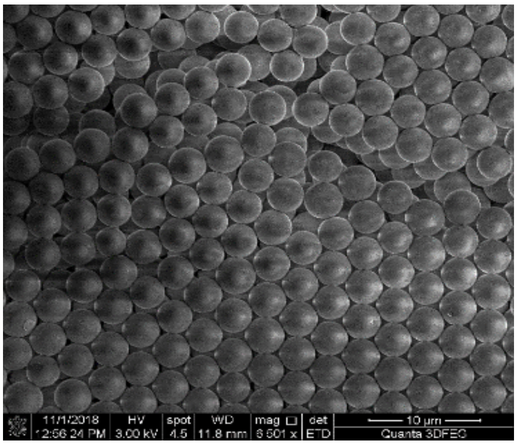

Figure S1. SEM image of purchased 3 µm silica particles.

S2. Synthesis of small unilamellar vesicles

Small unilamellar vesicles (SUVs) of 1,2-dioleoyl-*sn*-glycero-3-phosphocholine (DOPC) or 1,2-dipalmitoyl-*sn*-glycero-3-phosphocholine (DPPC) (Avanti Polar lipids, USA) were formed via thin film hydration and extrusion method. Specifically, 20 mM stock solutions of each lipid type were prepared in chloroform and aliquoted into 15 mL falcon tubes where the solution was spread into a thin film via vortexing and drying with a nitrogen (N_2) stream. After continued drying with N_2 for > 2 hrs, the thin film was rehydrated for a final lipid concentration of 1 mM using a buffer of 10 mM HEPES (4-2-hydroxyethyl-1-piperazineethanesulfonic acid) and 50 mM NaCl at 20 °C (DOPC) and 60 °C (DPPC) to ensure hydration above the melting temperature of the primary lipid. The buffer was prepared using ultrapure water (18.2 mΩ·cm) to a pH 7.4 and filtered with a 200 nm filter. After 2 min of vortexing, the resulting vesicles were extruded using an Avanti Mini Extruder (Avanti Polar Lipids, USA) 10 times with a 200 nm filter, and 11 times with a 100 nm filter, while maintained above T_m. The final concentration of the SUVs was 1 mM for confocal experiments, and 8 mM for calorimetric studies.

During vesicle preparation, specifically thin film preparation, PEGylated lipid and dye conjugated lipids components were added. To provide fluorescence to the bilayer, 0.25 mol% of dye-conjugated phospholipid was included. Specifically, 1,2-dioleoyl-*sn*-glycero-3-phosphoethanolamine-N-(lissamine rhodamine B sulfonyl) (DOPE–rhod) for DOPC, and 1,2-dipalmitoyl-*sn*-glycero-3-phosphoethanolamine-N-(lissamine rhodamine B sulfonyl) (DPPE–

rhod) for DPPC were used. The membrane additives 1,2-distearoyl-*sn*-glycero-3-phosphoethanolamine-N-[methoxy(polyethylene glycol)-2000] (ammonium salt) (DSPE–PEG_{2000}), 1,2-dioleoyl-*sn*-glycero-3-phosphoethanolamine-N-[methoxy(polyethylene glycol)-2000] (ammonium salt) (DOPE–PEG_{2000}) were also incorporated when forming the thin film at concentrations of 5% or 10% where necessary. All SUVs were kept a maximum of 3 days at 4 °C used to create CSLBs within 2 days of vesicle synthesis.

S3. Formation of colloid supported lipid bilayers (CSLBs)

CSLBs were formed by first mixing 940 μL of 10 mM HEPES 50 mM NaCl with 60 μL of 50 mg/mL washed 3 μm silica particles and 360 μL of 1 mM solutions of SUVs at 20 °C (DOPC) and 60 °C (DPPC) to ensure deposition above the T_m of the phospholipids. The suspension was mixed rapidly for 1 h after which the CSLBs were washed 5 times with fresh buffer via centrifugation and redispersion. Finally, the CSLBs were sonicated for 1 hour, followed by storage at 4 °C. All samples were used within one week.

S4. Florescence after photo bleaching (FRAP)

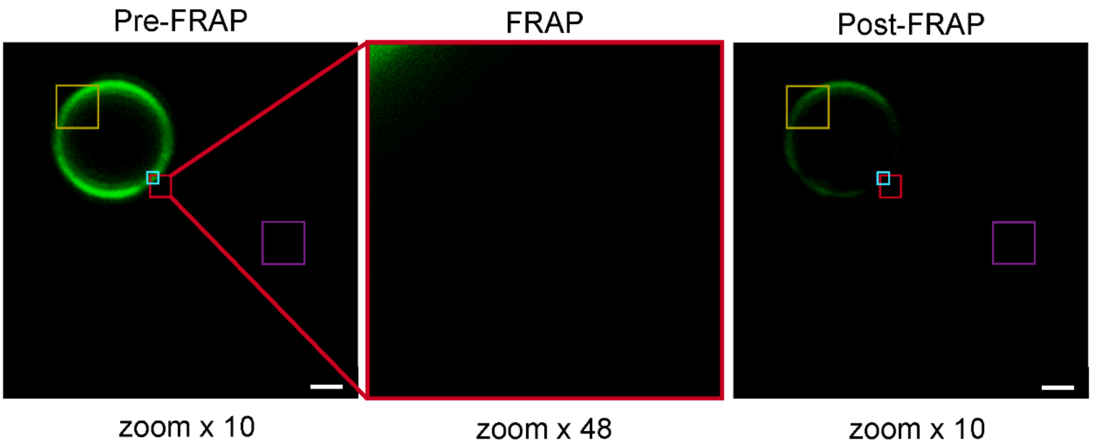

Figure S2. FRAP measurement was conducted by isolating a single CLSB particle in the left-hand corner of frame (left). FRAP occurred by isolating a small fraction of bilayer (red box) at 30 x 30-pixel ROI and zoomed in at 48 zoom. Once zoomed, laser intensity was increased to 100% for 2-3 frames (Middle). Laser intensity was than decreased to pre-FRAPed intensity and returned to original zoom factor and monitored for 10 minutes (right). FRAP analysis conducted on CSLB was conducted with the reference and background fluorescence of image with an 60 x 60-pixel area shown in yellow and purple respectively. The ROI monitored for FRAP recovery was 15 x 15-pixel area of the FRAP area of image shown in blue. Scale bar 1 μm.

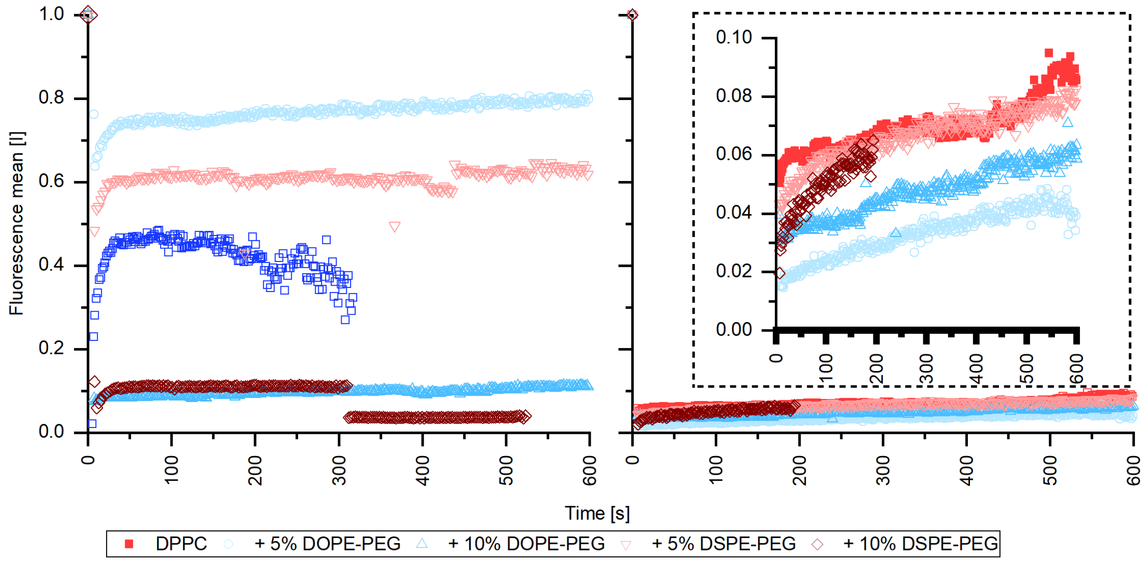

Figure S3. Full 10-minute scan of FRAP recovery of samples.

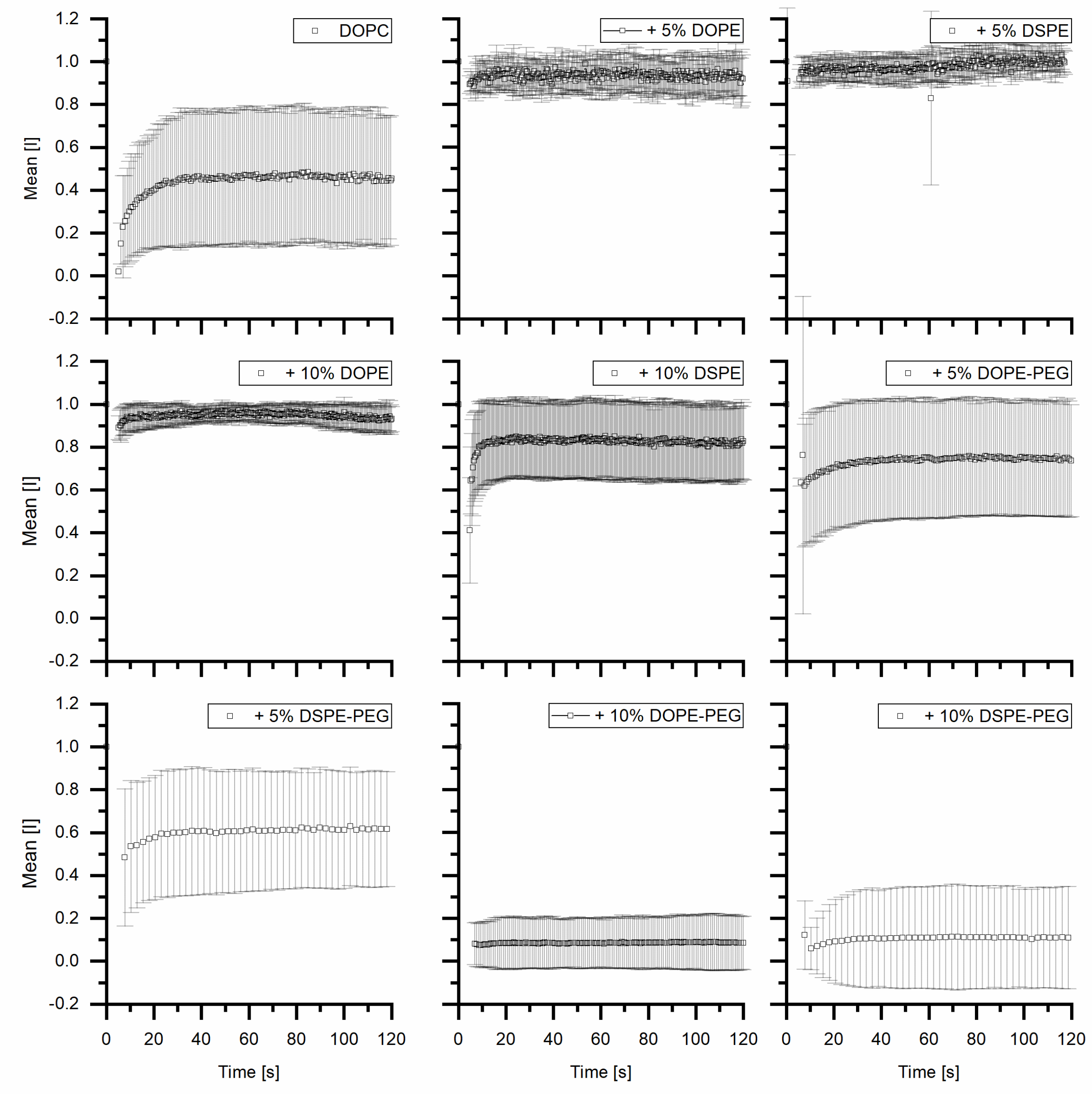

Figure S4. FRAP recovery curves of DOPC samples with standard deviation included (minimum *n*=9).

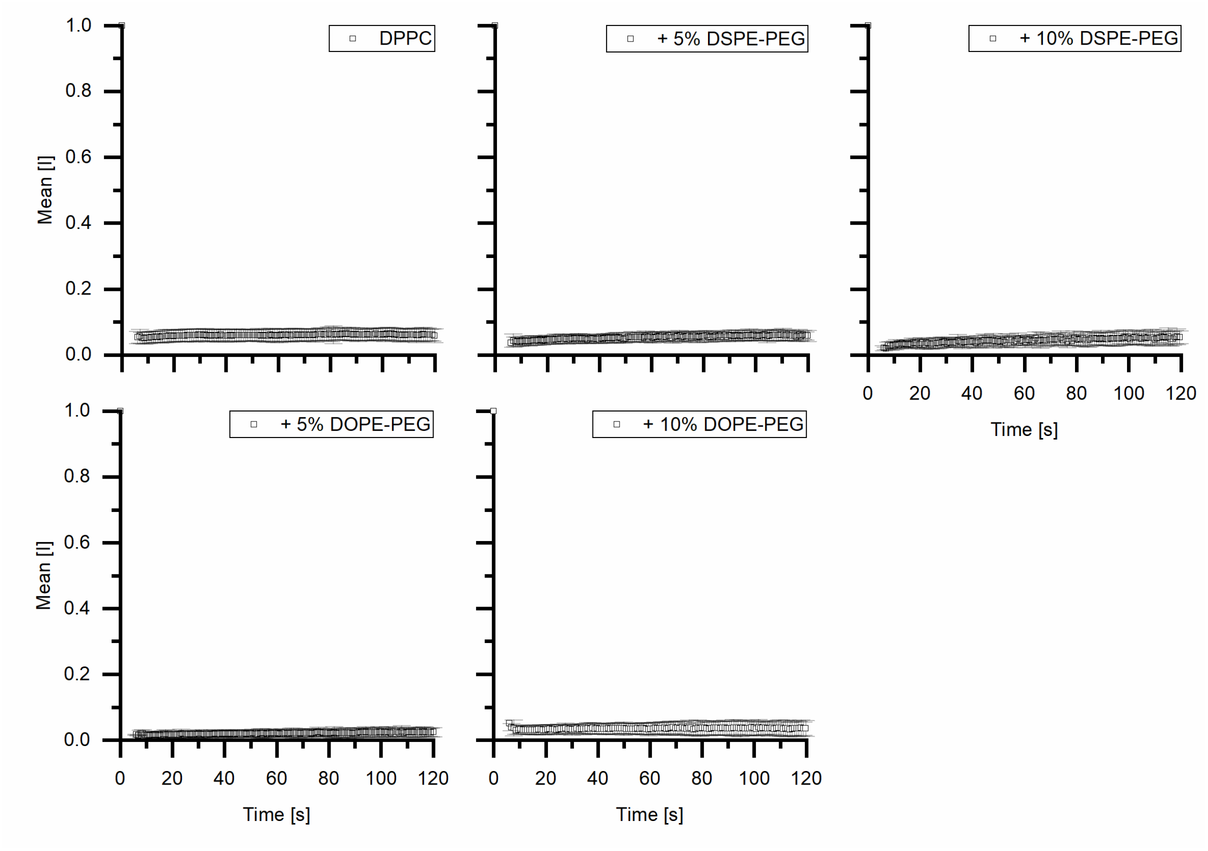

Figure S5. FRAP recovery curves of DPPC samples with standard deviation included (minimum *n*=9).

S6. Self-consistent mean-field theory (SCFT) predications of the composition of colloid supported lipid bilayers

The self-consistent mean-field theory (SCFT) employed to model phospholipid self-assembly follows the lattice discretization scheme by Scheutjens and Fleer.[1–3] SCFT is a predictive tool for self-assembly of amphiphiles and has been applied to systems ranging from block copolymers to surfactants.[4–6] The principle characteristic followed by SCFT to study self-assembly is the grand potential Ω, associated with inhomogeneities in the system of interest, held in equilibrium with the bulk solution.[7,8] SCFT is based upon Flory-Huggins (FH) theory, with first-order Markov chain statistics for the conformations of all the compounds involved.[9] Importantly, in order to optimize the free energy of the relative amounts of the compounds in solution, each chemical unit and their respective interactions are defined via the FH interaction parameters.

The equilibrium properties of self-assembled species are resolved by coupling SCFT with the thermodynamics of small systems.[7] A thermodynamically stable self-assembly is found

when the slope of Ω as a function of the aggregation number of a specified component k (number of phospholipids per bilayer cross-section, n_k^{exc}) decreases ($\partial\Omega/\partial n_k^{exc} < 0$). For phospholipid bilayers, when Ω=0 with $\partial\Omega/\partial n_k^{exc} < 0$, the bilayer is in equilibrium with the bulk phospholipids (at a volume fraction ϕ_k^{bulk}). At this condition, the chemical potential of lipids in bulk and in the self-assembled structure are equal. Furthermore, at this condition the equilibrium properties of the vesicle can be extracted. As we consider SCFT with concentration gradients in one direction in a planar lattice, the concentration profiles refer to the bilayer cross-section with the flat bilayer representing the vesicle bilayer composition. Furthermore, this explains why we look at the number of lipids per cross-section of vesicle as characteristic aggregation number.

SCFT is employed here to study the properties of vesicles resulting of mixing two different lipids, DOPC and DSPE–PEG_{2000}, hence following a co-assembly process. In this case, the impact that a given number n_2 of a second compound has on the Ω-n_1 curve of the single lipid case, is shown in Figure S6.

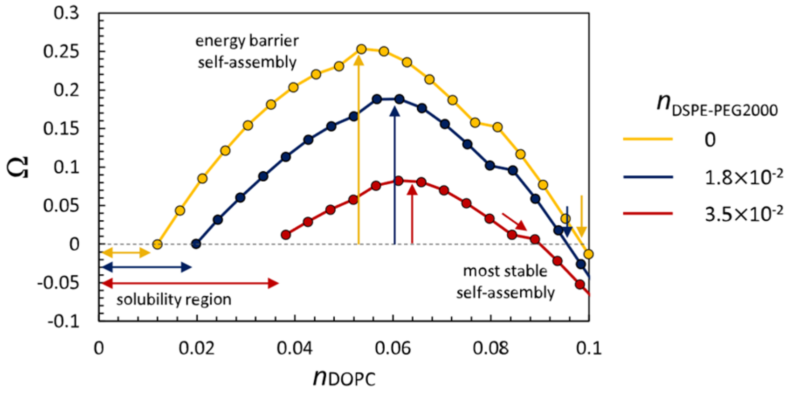

Figure S6. Grand potential Ω versus number of DOPC surfactants n_{DOPC} with increasing membrane additive DSPE-PEG_{2000} ($n_{DSPE-PEG_{2000}}$) as calculated by SCFT.

The most probable micelle is the one which satisfies $\Omega = 0$ with $\partial\Omega/\partial n_1^{exc} < 0$. For both DOPC alone and mixed with DSPE–PEG_{2000}, at low n_{DOPC} values the lipids do not self-assemble, as a solubility region (Figure S7) indicating all phospholipids dissolve in the bulk. Additionally, this region increases with increasing $n_{DSPE-PEG_{2000}}$. Upon increasing the number of DOPC molecules in the system, Ω first increases and then decreases with the maximum point relating to the minimum energy required for bilayer formation. Interestingly, this energy requirement decreases with increasing $n_{DSPE-PEG_{2000}}$. The addition of DSPE–PEG_{2000} also correlates with the shift of the peak to higher n_{DOPC}. The calculations are conducted using

mirror boundary conditions at the lower ($z = 0$) and upper bounds ($z = N_{lat}$) of the lattice; z the location parameter denotes the lattice coordinate and N_{lat} the total number of lattice layers used. In the case of CSLBs the presence of the silica (Si) surface it is assumed that the bulk concentration of micelles is that of the Si–free case and we calculate the properties of SUVs near a hard Si surface that minimize the free energy of the lattice. The surface of the Si colloidal particles is modelled as a negatively charged surface, with surface charge density of $200 mC/m^2$ impenetrable for all other compounds in the system (both phospholipids and solvent).[10] As shown in Figure 4 in the main manuscript, the phospholipid concentration profiles in the presence or absence of the Si surface are fairly close when fixing the Si–free lipid bulk concentration. In these computations, a mirror boundary condition is still present at $z = N_{lat}$.

The coarse-graining of SUV-forming phospholipids into beads of chemical units required for modelling on a lattice was based upon previous work on bilayer interactions and surfactant self-assembly.[5,11] Each chemical unit is considered as taking up a single lattice unit, except for the phosphate group which takes five units arranged in a cross-like shape (Figure S7). To account for electrostatic interactions, the lattice site size *b* needs to be specified, as well as the relative permeabilities ϵ of the different chemical units considered. Here we set $b = 0.3$ nm. All computations are conducted at a fixed salt concentration of 60 mM, corresponding to a fixed bulk counter-ion concentration of $\phi_{bulk}^{Na^+} \approx 0.001$ of the bulk solution. Further, electroneutrality of the total system is imposed. Finally, the lattice coordination number is set to $Z = 6$: we only consider a simple cubic lattice and conduct our calculations accounting for concentration gradients in one direction.[12] Hence all local concentration profiles of the molecules involved are allowed to vary in the *z*-direction, while the concentrations in other directions are identical. The set of molecular parameters is schematized in Figure S7.

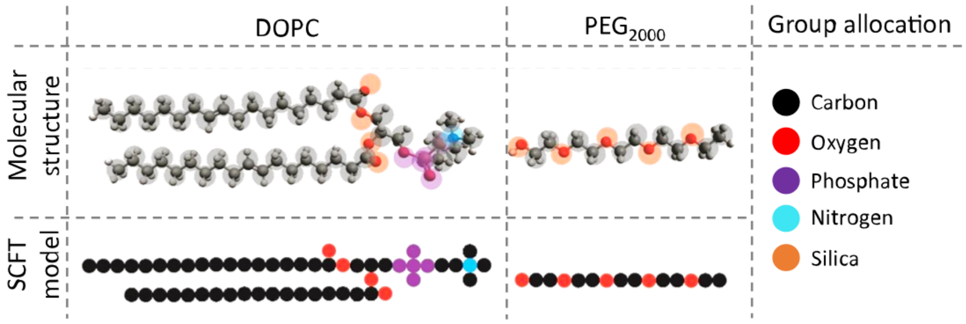

Figure S7. SCFT coarse-graining of the molecular structures of DOPC and short PEG_{2000} chains; groups presented in the rightmost column.

Below we provided the modelling parameters used for the SCFT computations. In Table S1 the FH interaction parameters χ_{ij}, relative permeabilities ϵ and number of charges per monomer considered for the different chemical units defined as lattice beads. We also provide the SCFT phospholipid structures used, as it further clarifies the coarse-graining conducted. Square brackets hold for branched segments from a central one. For the structures of the different gemini surfactants given as input in the SCFT computations, we exploit the `alias' feature of the sfbox. The input below corresponds to the case of two phospholipids in presence of free PEG. The charge of Nb is Nb=0 where Nb is the nitrogen group of the uncharged lipid.

//polymer composition

alias : tail : value : (C)17(C)1[(O)1](O)1(C)1(C)1[(O)1(C)1[(O)1](C)17](C)1 //tails are common

alias : headDOPC : value : (PO)1(PO)1[(PO)1][(PO)1](PO)1(C)2(N)1[(C)1][(C)1](C)1

alias : headDSPE : value : (PO)1(PO)1[(PO)1][(PO)1](PO)1(C)2(Nb)1[(C)1][(C)1](C)1

alias : PEO : value : (O)1(C)2

mol : DOPC : composition : (@tail)(@ headDOPC)

mol : PEGDSPE: composition : (@tail)(@ headDSPE)(C)1[(O)1](@PEO)45

mol : DPL : composition : (@PEOFree)2270

Table S1. Different chemical units modelled as lattice beads in SCFT as well as their Flory–Huggins interaction parameter χ_{ij}, relative permeabilities ϵ and charge per bead ν. All beads occupy a single lattice site, except for PO which takes 5 lattice units in a cross-like shape. The two values of N arise from the fact that in DOPC N is positively charged, while in DSPE–PEG$_{2000}$ it is neutralized.

		**H_2O**	**C**	**O**	**PO**	**N**	**Na**	**Cl**	**Si**
ϵ		80	2	1.5	80	7	10	10	5
ν/mon		0	0	0	-0.2	{1,0}	1	-1	-0.1
χ_{ij}	**H_2O**	0	1.6	-0.7	-1	0.5	0	0	1.0
	**C**		0	1.6	2	2	1.6	1.6	0
	**O**			0	0	0	-0.7	-0.7	0
	**PO**				0	0	-1	-1	0
	**N**					0	0.5	0.5	0
	**Na**						0	0	1.0

	**Cl**							0	1.0
	**Si**								0

The software sfbox was used to perform SCFT computations. sfbox input files and templates are available from dr. Álvaro González García upon reasonable request, as well as the Mathematica scripts used in the data processing of the output files.[13]

Below we provide more detailed SCFT results on the phospholipid bilayers used to model the vesicles studied experimentally. In Table S2 we summarize several equilibrium properties of the systems studied, as computed using SCFT.

Table S2. SCFT computed equilibrium properties of self-assembled SUVs modelled as bilayers from the unimers specified: aggregation number n^{exc}, bulk unimer concentration (SCFT-provided cmc, ϕ^{bulk}), and gyration (D_g) and hydrodynamic (D_h) diameters.

**Lipid**	n^{exc}	ϕ^{bulk}=ϕ^{CMC}	D_g **[nm]**	D_h **[nm]**
DOPC	0.10	$2.83 \cdot 10^{-6}$	3.0	2.8
DSPE	0.09	$1.12 \cdot 10^{-5}$	2.7	2.1
DSPE–PEG_{2000}	0.02	$3.58 \cdot 10^{-2}$	9.6	11.4

With increasing tail length, surfactants become more insoluble and therefore their bulk concentration at vesicle–lipid equilibrium ϕ_k^{bulk} decreases (the vesicle–lipid equilibrium shifts towards the vesicle). Note that the density of surfactants per cross section is roughly constant for all vesicle-forming surfactants, whilst it dramatically decreases upon PEGylation of DOPC (leading to DSPE–PEG_{2000}). This follows from the fact that PEG is water-soluble, therefore the bilayer–bulk equilibrium shifts to the bulk upon PEGylation.

The thermodynamics of mixed lipid vesicle formation are summarized in Figure S5 for the lipid mixture of interest: DOPC with added DSPE–PEG_{2000}. We express the amount of DSPE–PEG_{2000} present in the lipid mixture in terms of its aggregation number as $\alpha = n_{DOPC}^{exc}/n_{tot}^{exc}$, with $n_{tot}^{exc} = n_{DOPC}^{exc} + n_{DSPE-PEG_{2000}}^{exc}$. As observed in Figure S8a, the amount of DOPC in the self-assembly decreases with respect to that of the pure DOPC vesicle. Overall, DSPE–PEG_{2000} phospholipids gradually replace DOPC phospholipids upon increasing α. At $\alpha = 0.6$ (60% DSPE–PEG_{2000}) however more than 40% of the DOPC phospholipids are displaced in the

bilayers. This can be explained by the fact that the PEGylated phospholipids occupy more space in the bilayer. Contrary to self-assemblies formed from a single lipid, this decrease in the n_{DOPC}^{exc} is accompanied by a decrease in CMC/bulk concentration (Figure S8b), yet the total lipid bulk concentration increases with increasing α due to the high solubility of DSPE–PEG$_{2000}$. The energy gain per diblock upon self-assembly (Figure S8c, $\Delta G/n_{tot}^{exc}$) increases with increasing α: there is a significant energy gain per diblock upon self-assembly of more DSPE–PEG$_{2000}$ lipids. Note that, as in experiments only (5–10) % of the lipids present in the mix are DSPE–PEG$_{2000}$, we focus on the regions indicated by dashed boxes. From Figure S8, we conclude that a minute addition of DSPE–PEG$_{2000}$ is sufficient to dramatically alter the equilibrium properties of the DOPC-forming SUVs, therefore eventually also their interaction.

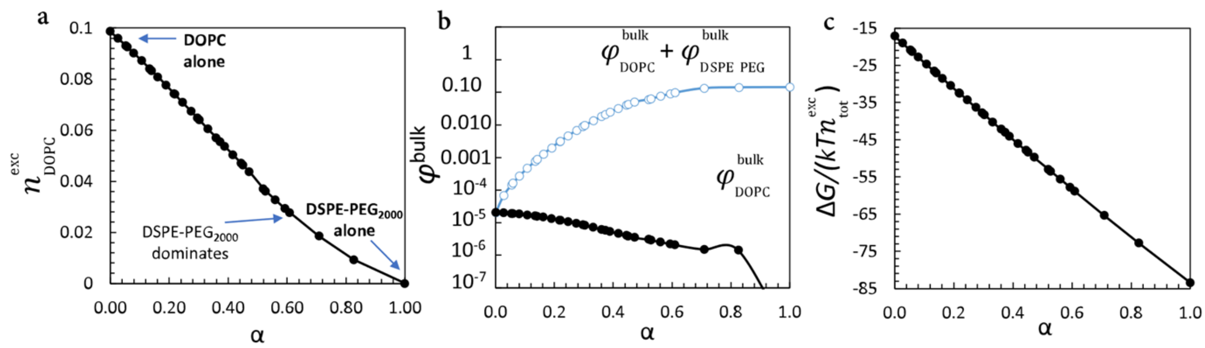

Figure S8. Equilibrium properties of SUVs emerging from mixing DOPC and DSPE-PEG$_{2000}$ lipids at the indicated ratios where $\alpha = n_{DSPE-PEG_{2000}}^{exc}/(n_{DOPC}^{exc} + n_{DSPE-PEG_{2000}}^{exc})$ a) aggregation number change in DOPC, b) variation on the lipid bulk concentration, c) energy gain per lipid upon self-assembly.

Illustrative volume fraction profiles of DOPC+DSPE–PEG$_{2000}$ vesicles are presented in Figure 4a of the main manuscript. The PO and N groups are symmetrically distributed around the maximum lipid volume fraction, both when considering DOPC alone and mixed with DSPE–PEG$_{2000}$. Naturally, with increasing $n_{DSPE-PEG_{2000}}^{exc}$ the vesicle composition contains more DSPE–PEG$_{2000}$, reflected in its overall increasing volume fraction profile in and near the bilayer region.

The presence of the Si surface affects the vesicle concentration profiles based on the type of phospholipids involved (Figure 4b). In case of the DOPC phospholipids, the nitrogen group *N* is charged. Therefore, it adheres to the Si–surface, electrostatically anchoring the vesicle to the Si–surface and causing the strongest deviation observed between the profiles with and without the Si surface. The N group of the DSPE phospholipid is however neutralized, therefore there is mainly steric and electrostatic repulsion between the Si–surface and in the

phospholipid vesicle. The DSPE vesicle is formed further apart from the Si–surface than the DOPC one, and the overall (black) profiles in presence or absence of the Si surface are rather close (due to the lack of an anchor). For the PEGylated lipid DSPE–PEG_{2000}, depletion of the PEG chains from the Si–surface leads to a bilayer formed far enough from the Si surface so the configurational entropy loss of these chains is minimized. This bilayer is not deformed compared to the Si-free case (main manuscript, Figure 4). In absence of a Si-surface, the N and PO groups as well as PEG chains of DSPE–PEG_{2000} are distributed symmetrically around the maximum DOPC volume fraction (Figure 4a). Due to the high entropic penalty of PEG chains near the Si surface, this symmetric distribution is broken in the case of a silica surface, and all groups of DSPE–PEG_{2000} prefer to be located towards the solution. As observed for the Si-free case, there are no significant changes in the distribution of DOPC molecules (Figure 4a). Variations are appreciable only when $\phi_{DOPC}^{bulk} \ll \phi_{DSPE-PEG_{2000}}^{bulk}$. Particularly, changes are only appreciable in the outer peripheral domain of the mixed bilayer: it appears the N and PO groups of DSPE–PEG_{2000} are absorbed into the SUV while the PEG chains are not only depleted from the Si surface but are also located outside the DOPC rich bilayer.

S6. Atomic force microscopy on planar supported lipid bilayers

Circular mica substrates glued on Teflon discs were used for the atomic force microscopy (AFM) experiments. SLBs were prepared by depositing 90 µL of 1 mM SUV dispersion onto a freshly cleaved mica. After incubation above the lipid phase transition temperature for 30 minutes, the samples were rinsed several times with buffer solution, preventing from having SUVs unfused onto the surface.

The AFM topographical images were obtained with an MFP-3D atomic force microscope (Asylum Research) using V-shaped SNL silicon nitride cantilevers (Bruker AFM Probes) with a nominal spring constant of 0.35 N/m. All AFM topographies were acquired with AC mode, at room temperature and under liquid conditions.

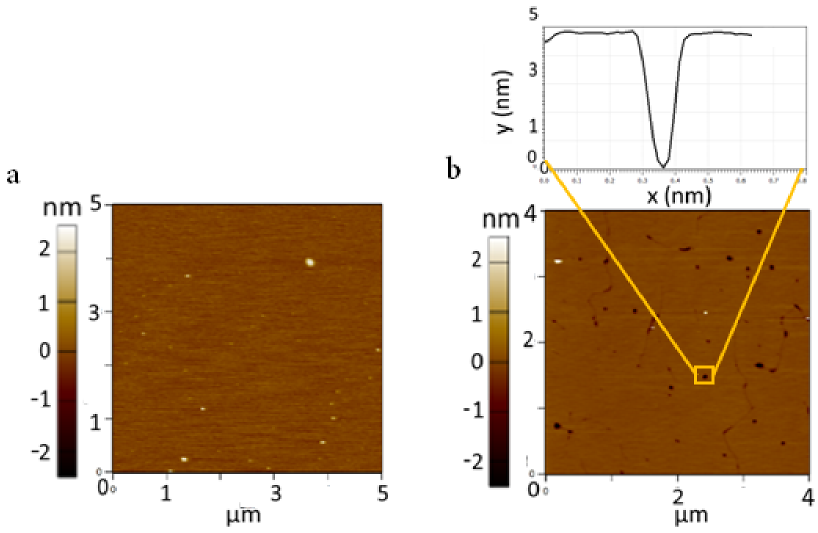

Figure S9. AFM topography and profile of planar supported lipid bilayers at room temperature of a) DOPC and b) DPPC.

S7. Differential Scanning Calorimetry

Calorimetric studies were carried out using a Multi-Cell micro-DSC (TA Instruments). Samples (~8 mM of SUV or CSLB) were dispensed into pre-calibrated sample cells, cell 1 and cell 2, with an approximant weight of 400 mg. Buffer solution (~400 mg) was added to cell 3, allowing heating and cooling scans of samples with buffer to be completed simultaneously. Additionally, cell 4 remained empty for reference of the crucible. A scan heating rate and cooling rate of 0.5 °C /min and 1 °C /min were used for all samples, respectively.

Sample runs were performed in the temperature range between 10 °C and 80 °C and repeated in technical triplicate to ensure the closeness of successive measurements within the same conditions. Each heating and cooling scan was than examined using NanoAnalyze Data Analysis (Version 3.11.0, www.tainstruments.com) to determine the accuracy of the measured value (T_m) and true value reported in literature (Figure S10).

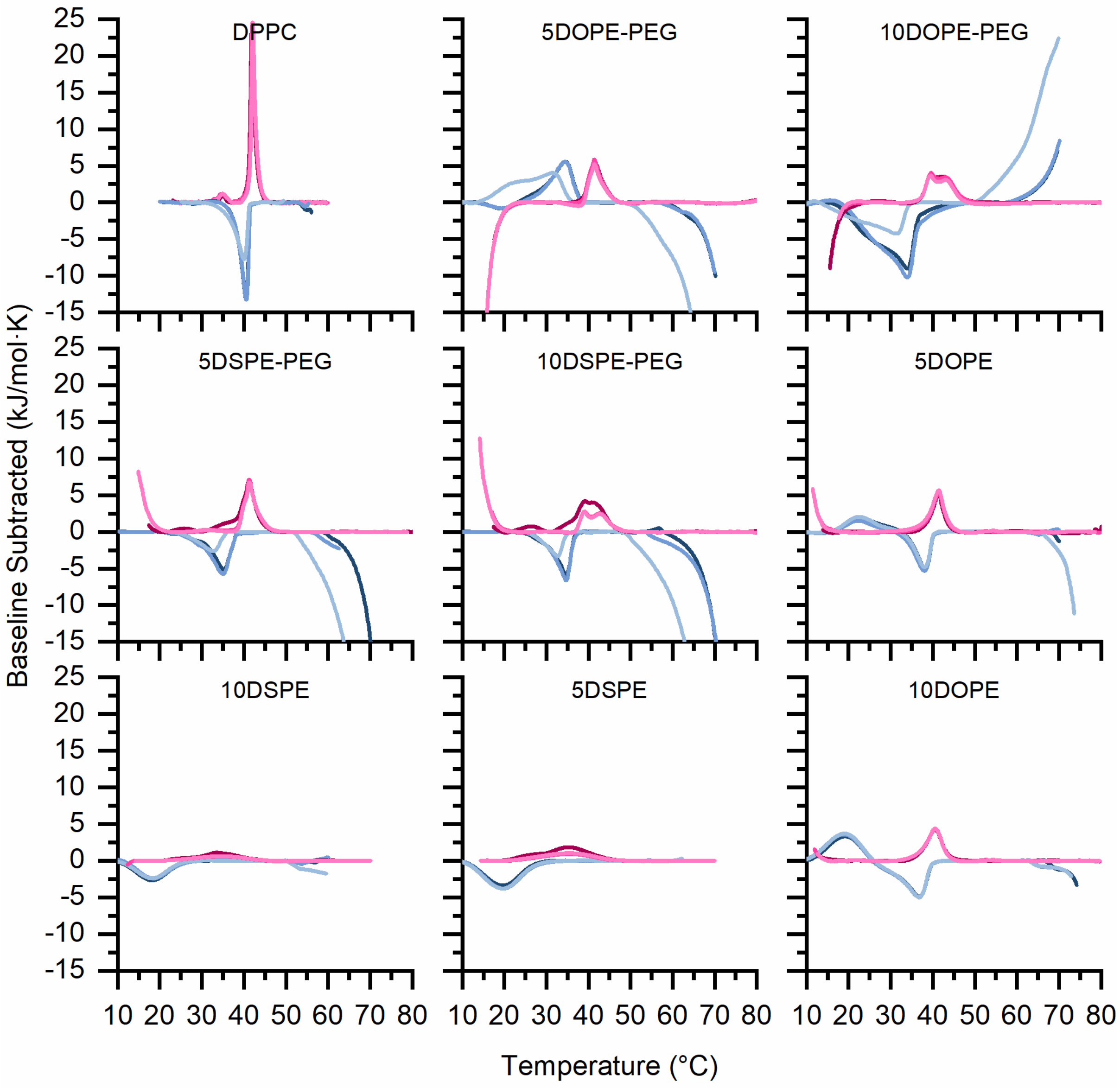

Figure S10. DSC thermograms heating (red) and cooling scans (blue) of DPPC SUVs with 0, 5 and 10 mol%; DOPE–PEG_{2000} , DSPE–PEG_{2000} , DSPE and DOPE incorporated into the membrane. DSC thermograms are of background subtracted raw data.

Initially, the background was subtracted from raw sample data in µJ/s and converted to molar heat capacity by volumes (moles). In order to exclude initial heating/ cooling ramps (artifacts introduced by instrument), analysis on data was completed between the ranges of 25 °C and 55 °C were selected for baseline correction (20-60 °C heating, for DPPC only). The baseline of raw data was constructed via sigmoidal baseline fitting, followed by Gaussian fitting models to ascertain T_m and FWHM. Models were fitted for a maximum 100000 iterations with a fitting precision of 1×10^{-6} and statistics completed at 1000 trails at 95% confidence level.

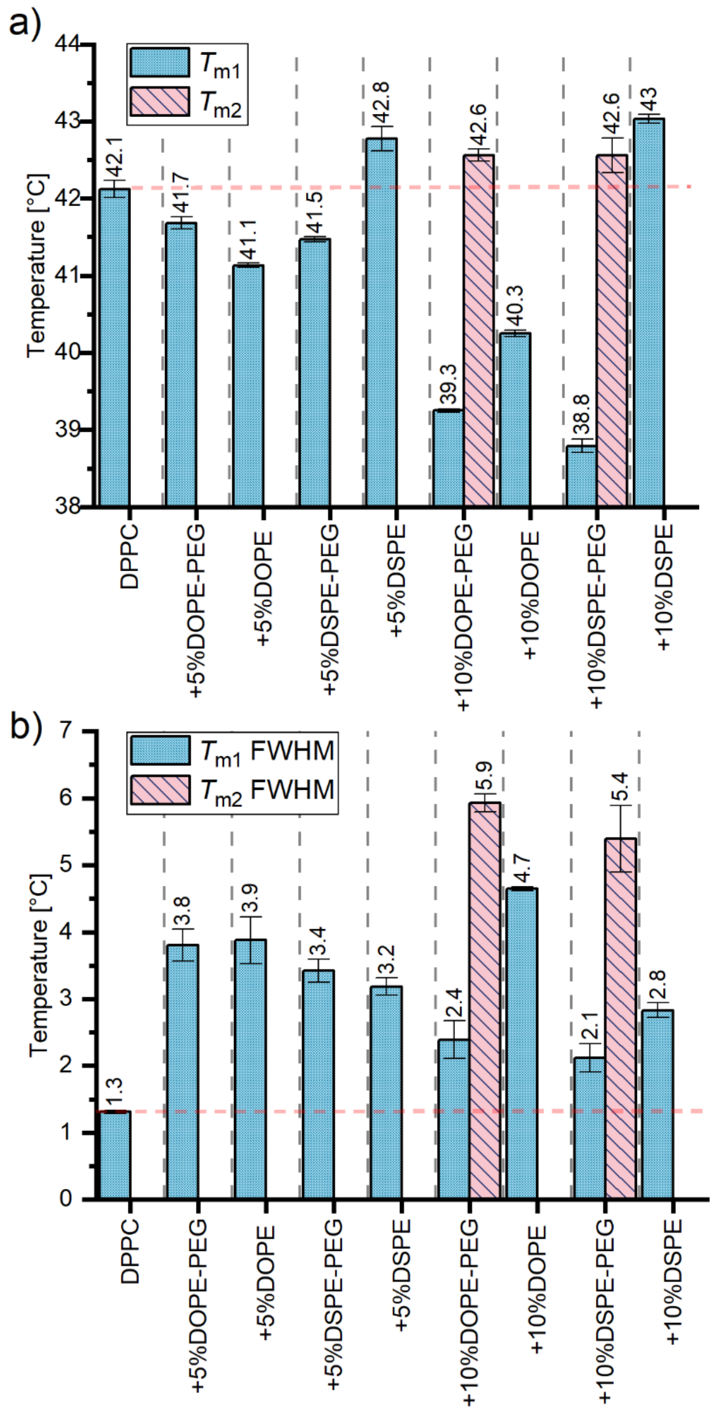

Figure S11. a) Average T_m of CSLB samples and b) Average full width half maximum of T_m peaks of DPPC SUVs with 5 and 10 mol%; DSPE, DSPE–PEG$_{2000}$,DOPE and DOPE–PEG$_{2000}$ incorporated into the membrane. (n=3)

S8. Dynamic light scattering

All light scattering measurements were performed on an ALV/ CGS-3 MD-4 multi detection goniometer system, with a 400 mW argon laser operating at a wavelength of 532 nm. Samples were measured 4 times for 5 seconds at 90°. The second order autocorrelation functions of samples were processed by an ALV-7004 Digital Multiple Tau Real Time Correlator. The first order autocorrelation functions were analyzed with AfterALV program (AfterALV 1.0d, Dullware) using the CONTIN algorithm (inverse Laplacian transformation of the field autocorrelation function) to calculate the decay rate, of the particles. The data was transferred to CSV files for further analysis in Origin (Origin 2019 (Academic), OriginLab Co, Northampton, MA, USA).

All measurements were conducted at 20 °C in 1 cm path-length borosilicate disposable tubes. All solvents were filtered using a 0.2 µm filter. For each measurement the viscosity was corrected for any change in temperature during measurements.

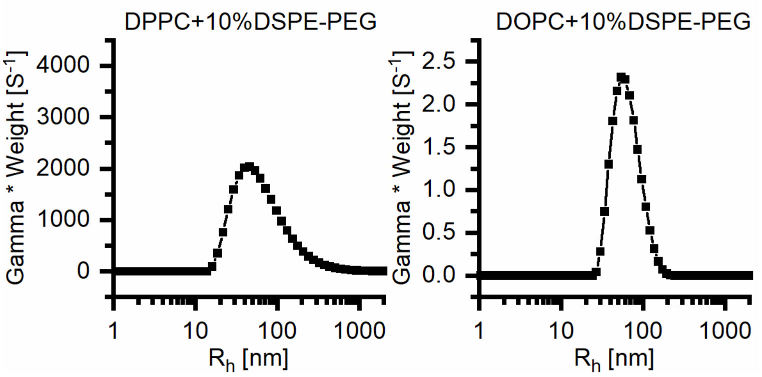

Figure S12. Dynamic light scattering curves at 90° of DPPC and DOPC SUV samples with 10%DSPE-PEG incorporated into membrane.

S9. References

[1] J. M. H. M. Scheutjens, G. J. Fleer, *J. Phys. Chem.* **1979**, *83*, 1619–1635.

[2] J. M. H. M. Scheutjens, G. J. Fleer, *J. Phys. Chem.* **1980**, *84*, 178–190.

[3] G. Fleer, M. . Cohen Stuart, J. M. H. . Scheutjens, T. Cosgrove, B. Vincent, *Polymers at Interfaces*, Chapman & Hall, London, **1998**.

[4] F. Li, A. T. M. Marcelis, E. J. R. Sudholter, M. A. Cohen Stuart, F. A. M. Leermakers, *Soft Matter* **2009**, *5*, 4173.

[5] Á. González García, E. . Timmers, N. Romijn, S. Song, S. Sahebali, R. Tuinier, I. K. Voets, *Colloids Surfaces A* **2019**, *561*, 201–208.

[6] A. Ianiro, J. Patterson, Á. González García, M. M. . van Rijt, M. M. R. . Hendrix, N. A. J. . Sommerdijk, I. K. Voets, A. C. C. Esteves, R. Tuinier, *J. Polym. Sci. Part B* **2018**, *56*, 330–339.

[7] F. A. M. Leermakers, J. C. Eriksson, H. Lyklema, in *Fundam. Interface Colloid Sci. Soft Colloids*, Elsevier, **2005**.

[8] Á. González García, A. Ianiro, R. Beljon, F. A. M. Leermakers, R. Tuinier, *Soft Matter* **2020**, *16*, 1560–1571.

[9] P. . Flory, *Principles of Polymer Chemistry*, Cornell University Press, **1953**.

[10] E. Guzman, L. Fernandez-Pena, G. . Luengo, A. . Rubio, A. Rey, F. Leonforte, *Polymers (Basel).* **2020**, *12*.

[11] L. A. Meijer, F. A. M. Leermakers, J. Lyklema, *J. Phys. Chem. B* **1995**, *99*, 1782–17293.

[12] Á. González García, M. M. . Nagelkerke, R. Tuinier, M. Vis, *Adv. Colloid Interface Sci.* **2020**, *275*.

[13] “Wolfram Research Inc. Mathematica,” **n.d.**

[14] L. Redondo-Morata, M. I. Giannotti, F. Sanz, *Langmuir* **2012**, *28*, 12851–12860.
